# GelPOINT single-port laparoscopy-assisted transanal minimum invasive surgery for low rectal cancer: a preliminary report on the use of the GOD VISION wireless smart glass-shaped monitor

**DOI:** 10.1186/s12957-020-01924-6

**Published:** 2020-06-26

**Authors:** Nobuhisa Matsuhashi, Yoshinori Iwata, Mana Kawajiri, Takao Takahashi, Shigeru Kiyama, Shunya Kiriyama, Machi Uehara, Takeharu Imai, Hisashi Imai, Yoshihiro Tanaka, Naoki Okumura, Kazuhiro Yoshida

**Affiliations:** 1 Department of Surgical Oncology, Gifu University Hospital, 1-1 Yanagido, Gifu City, Gifu 501-1194 Japan; 2 General and Cardiothoracic Surgery, Gifu University Hospital, 1-1 Yanagido, Gifu City, Gifu 501-1194 Japan

## Abstract

**Background:**

The GOD VISION wireless smart glass-shaped monitor (INBYTE) was used in the treatment of an elderly patient with mixed breathing disorder undergoing transanal minimally invasive surgery (TAMIS) for low rectal cancer under lumbar anesthesia.

**Method:**

After wearing the GOD VISION wireless smart glass-shaped monitor, we attached it to the Gel POINT Path® (Applied Medical). The tumor was surgically removed from all layers of the rectum using an ENDOPATH Electrosurgery PROBE PLUS II System® (a spatula-type electric scalpel) and the site was closed after sufficient washing.

**Results:**

The total operation time was 93 min, and the estimated blood loss was 6 mL. The patient was discharged without complications on postoperative day 14. No local recurrence or distant metastasis in the 7 months after the operation. The patient remained in a good condition with the preservation of the anal function.

**Conclusions:**

It is necessary to accumulate cases and to perform long-term follow-up. In addition, the anal side operators are able to operate without discomfort. In the present case, the GOD VISION wireless smart glass-shaped monitor allowed the TAMIS operation to be performed more comfortably.

## Introduction

The curative methods that are used in the treatment of early rectal cancer include endoscopic excision [1], local excision, and total mesorectal excision (TME) with the lymph node dissection. Buess et al. reported the performance of transanal endoscopic microsurgery (TEM) a transanal local resection [2, 3]. In recent years, Lacy and Sylla et al. reported that throughout the world, TaTME is considered as a surgical method for cases of rectal cancer in which surgical resection via by the conventional approach from the abdominal cavity is difficult, such as operations involving male patients, a narrow cavity, or a bulky tumor [4, 5]. However, it may be difficult to be performed TaTME in cases involving elderly patients or patients who have previously undergone surgery for rectal cancer. The operator often needs to be in an uncomfortable position in order to enter between the patient’s legs, in order to view the endoscope monitor. We introduced a procedure wherein the monitor is reflected in a smart glass by attaching a GOD VISION device. We believe that this device was useful for the operation.

## Case presentation

**Fig. 1:**
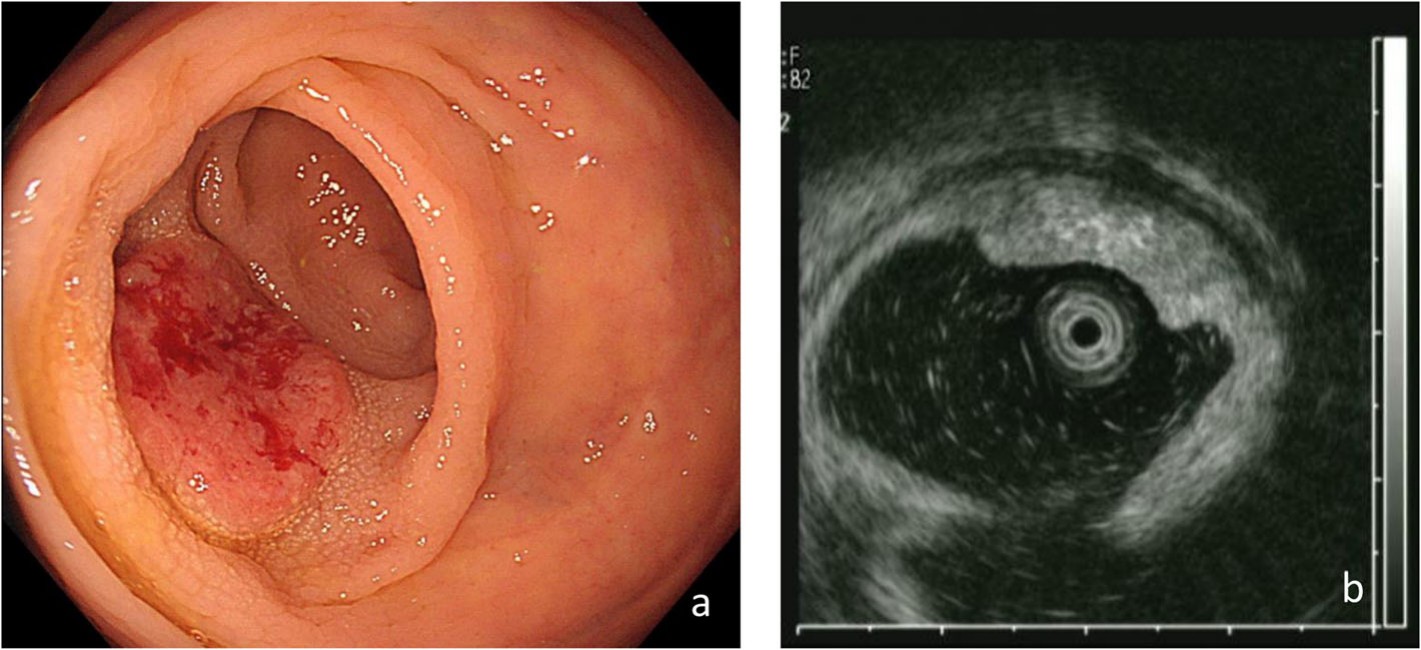
**a** Colonoendoscopy image revealing a rectal tumor at the left wall of lower rectum. **b** Colonoendoscopic ultrasonic view of the tumor demonstrating the presence of a invasive tumor with a high echo located in the third layer (muscular propria) of the rectum

An 82-year-old female patient with occult blood in a stool specimen was referred to Gifu University Hospital (Gifu, Japan) for evaluation and treatment. Colonoscopy revealed a superficial elevated tumor in the lower rectum (Rb) (Fig. 1a, b), and the examination of a biopsy specimen revealed adenocarcinoma. There were no signs of metastasis. The patient had a previous history of traumatic injury in a road traffic accident, which had resulted in right leg amputation at 80 years of age. She had been tracheotomized after the procedure and hospitalized with artificial respiration management for 7 months. Consequently, she had a chronic breathing disorder. A breathing function examination revealed mixed ventilation disorder, with the following values: %VC, 64.9%; FEV1 .0%, 50.7%; and FEV1, 0.72L.

The anesthesiologist judged that it would be impossible to perform general anesthesia due to her respiratory function.

Her medical history was also notable for diabetes, with insulin use and chronic atrial fibrillation, which was treated with an oral anti-coagulant. Based on the patient’s general condition, we decided that it was not appropriate to perform super low anterior resection with laparotomy and laparoscopic surgery. Instead, we performed transanal local excision, as it would be possible to perform with the patient breathing under lumbar anesthesia.

## Surgical technique

**Fig. 2:**
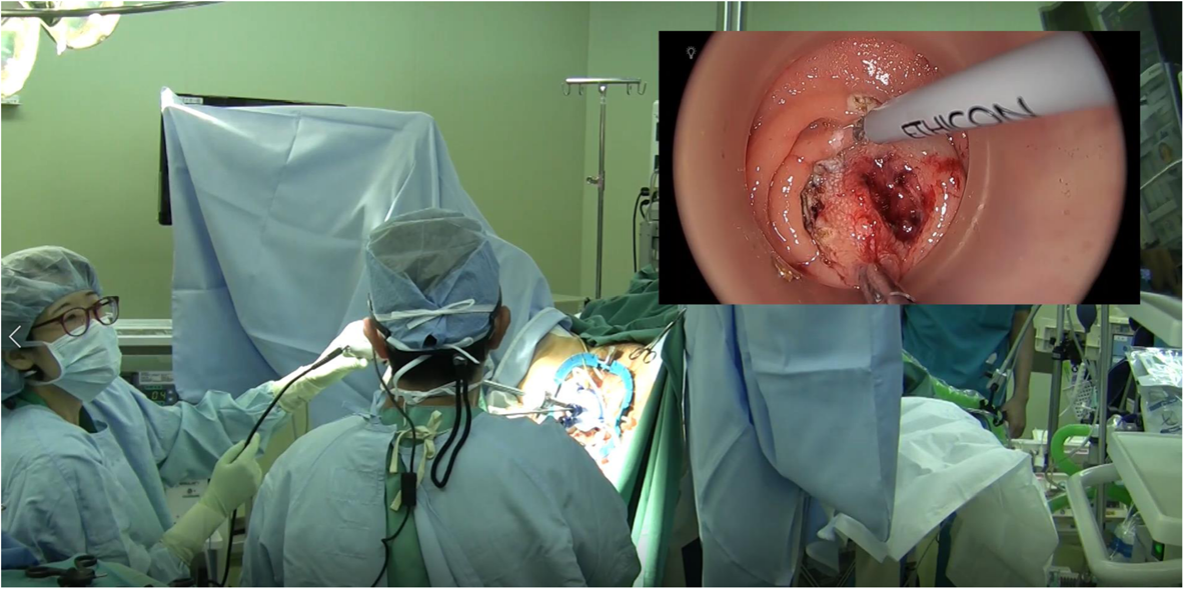
Intraoperative view of the patient anal side.Right upper side image: a glasses-shaped monitor

**Fig. 3:**
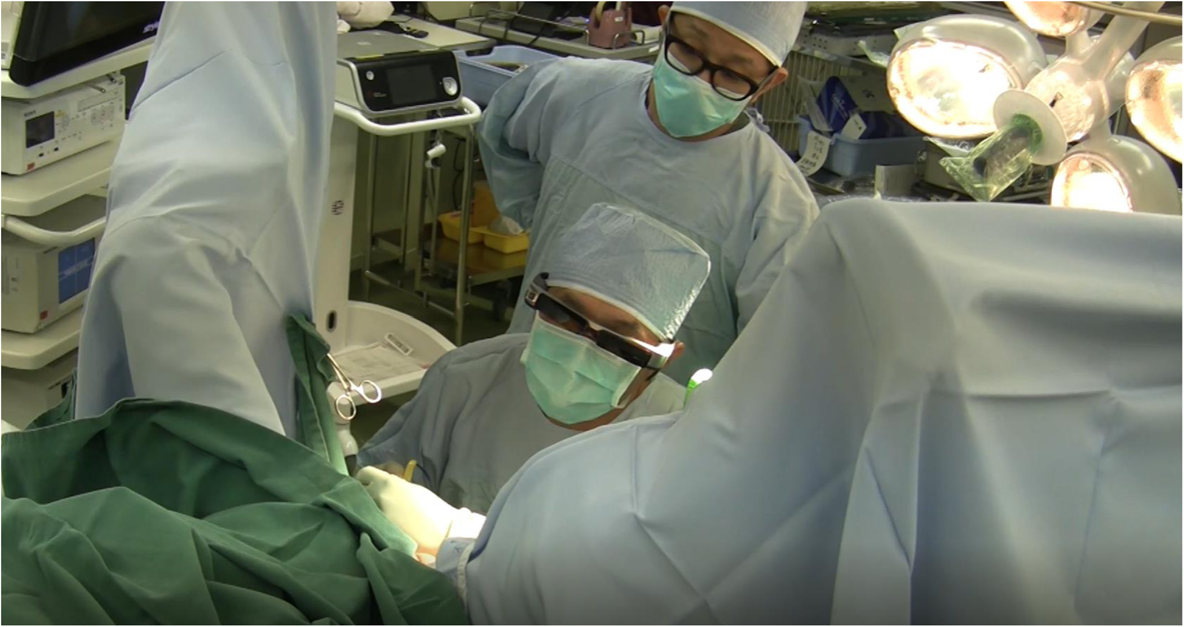
Intraoperative view from the patient head side

**Fig. 4:**
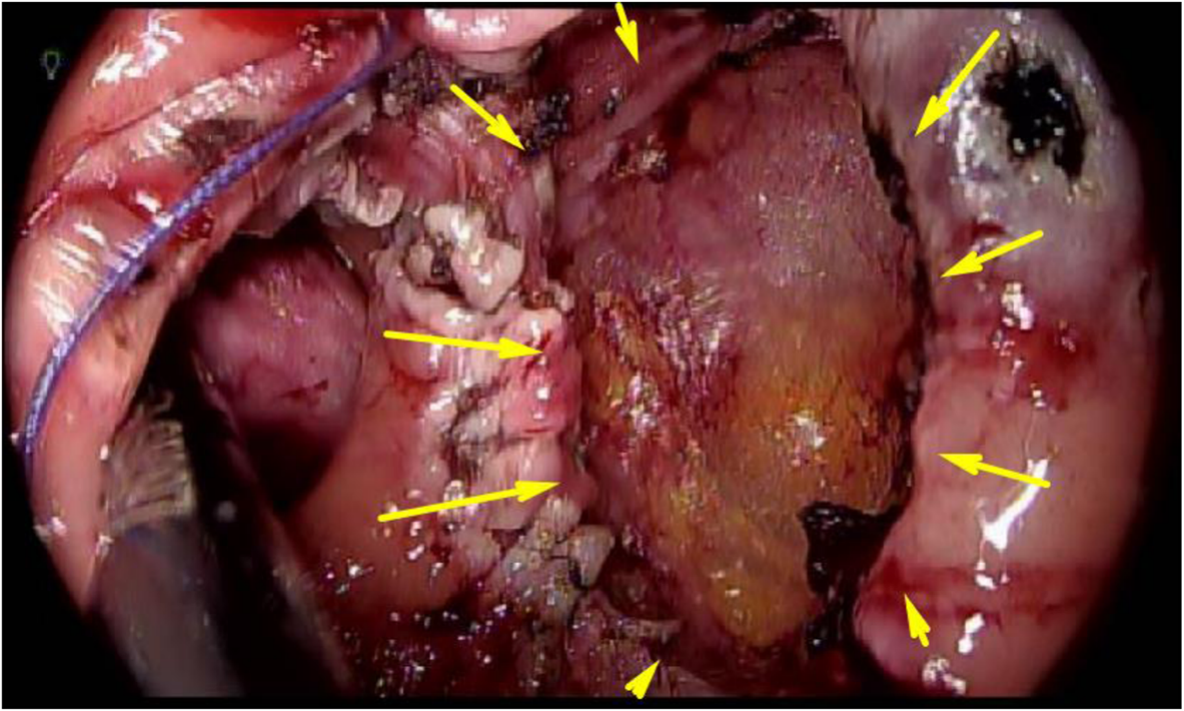
Transanal intraoperative view after tumor resection

The patient received mechanical bowel preparation (MBP) combined with oral antibiotics for preoperative bowel preparation. Lumbar anesthesia was used. After successful anesthesia, the patient was placed in the lithotomy position with her head lowered. Her lower limbs were raised and spread out, fully exposing the anus. Anal dilation was performed using a self-retaining anal retractor (Lone Star Retractor, Cooper Surgical, Trumbull, CT). A trans-access platform (GelPOINT Path Applied Medical, Rancho Santa Margarita, CA) was introduced for dissection of the distal portion of the tumor using TAMIS. A CRYSTAL VISION 450D (AMCO Medical Company) was used to maintain pneumorectum 15 mmHg with carbon dioxide. Conventional laparoscopic instruments were used. Dissection was carefully performed from the oral side to the anal side (Figs. 2 and 3). The operative procedures performed step by step on various sides to avoid injury to the tumor. The tumor was removed through the transanal access device (Fig. 4). Intraluminal lavage was performed with saline, and hemostasis was confirmed. A histopathological examination revealed the expression of TUB2 with a negative margin. The post-operative course was uneventful. The patient was discharged on the 14th postoperative day and is currently undergoing regular follow-up at 7 months after surgery without any signs of recurrence.

## Discussion

Transanal minimally invasive surgery (TAMIS) is used to treat early colorectal tumors. Endoscopic submucosal dissection (ESD) for the resection of tumors extending above the dentate line (particularly those with concomitant hemorrhoids) is technically difficult [6]. We presented a case involving a patient with lower rectal adenomacarcinoma extending above the dentate line, in which TAMIS was performed to achieve accurate excision and prevent complications.

The recently developed TaTME technique can be used to resect lesions completely and ensure a negative circumferential margin. According to the transanal endoscopic platform, TaTME can be classified as transanal endoscopic microsurgery-TME (TEM-TME) using a TEM platform and transanal minimally invasive surgery-TME (TAMISTME) using a TAMIS platform.

Early on, at the time of TaTME implementation, the more common approach was TEM-TME [7]. In 2010, the application of laparoscopy-assisted TEM-TME without postoperative complications was reported by Sylla et al. [4]. Along with the implementation and development of intraluminal minimally invasive surgery using the TAMIS platform, TAMIS was developed to achieve TaTME operations. Surgeons have now preferentially adopted disposable multi-channels single-port platforms for TAMIS use, such as the GelPoint (Applied Medical Company) [8, 9].

In general, the operator of the anal side can watch the endoscope picture on patient’s head-side monitor. But the operator often needs to be in an uncomfortable position in order to enter between the patient’s legs. Especially, the operators are forced into an uncomfortable position in order to view the endoscope monitor. In order to reduce the uncomfortable position, we performed the surgery transanal local excision with the GOD VISION system.

The GOD VISION system is characterized without turning toward the monitor and is able to maintain a comfortable posture. With recent innovations in surgery, new approaches to minimally invasive surgery using endoscopic surgery techniques have become mainstream. In laparoscopic surgery, it is necessary to move both hands freely while watching a surgical monitor. In TaTME and TAMIS, the operator positioned between the patient’s legs with the patient in the lithotomy position. New methods and medical equipment to reduce the patient’s burden are researched and developed every day [10, 11].

The GOD VISION system was developed as a glasses-shaped monitor worn by the operator that obviates the need to look at the monitor during laparoscopic surgery and which thereby reduces positional discomfort. The GOD VISION system displays video output from an endoscope video image processor. The output is transmitted to the GOD VISION by radio, where it is received by an onboard receiver and displayed. The weight of the wireless smart glass-shaped monitor is very light at 69 g.

Elements such as the effects of magnification and the view did not differ from a normal endoscopic monitor, with the image being of comparable size. We felt that we were able to perform the operation without trouble from the perspective and influenced by the appearance to accompany. In addition, wireless communication delay exits about 0.1 s in the first-generation of the GOD VISION. However, we do not feel a wireless communication delay in the present generation of the GOD VISION.

There are potential concerns related to the wearing of a smart glass device for a long time, including general sight fatigue, photosensory attack (photosensitive seizures [PSS]), and visually induced motion sickness (VIMS). The glass shaded the light at the time of smart glass wearing is not influenced by the appearance to accompany. It was considered that the influence was reduced, as we were able to concentrate on the operation field image. We considered that we did not have to worry about the development VIMS during the operative time of normal laparoscopic colorectal surgery because image disconformity (excessive time–space changes) was not excessive. However, when performing laparoscopic colorectal surgery, the head is often positioned at a low angle, toward the right and left. In the case of the operative procedure, it is often necessary to maintain the operation field while in a static condition in an uncomfortable position. TaTME often disturbs the field of vision when both legs watch a monitor and often operates from an uncomfortable position.

Thus, the use of the GOD VISION system could reduce the burden on the assistant of the operators.

## Conclusions

The GOD VISION wireless smart glass-shaped monitor (INBYTE) was applied in TAMIS for low rectal cancer in an elderly patient with mixed breathing disorder under lumbar anesthesia. Using the GOD VISION device, we were able to secure a field of vision to perform an operation continuously without discomfort.

## Data Availability

Not applicable
